# Draft Genome Sequence of a *Mycobacterium africanum* Clinical Isolate from Antioquia, Colombia

**DOI:** 10.1128/genomeA.00486-16

**Published:** 2016-06-02

**Authors:** U. A. Hurtado, J. S. Solano, A. Rodriguez, J. Robledo, F. Rouzaud

**Affiliations:** a Corporación para Investigaciones Biológicas (CIB), Medellín, Colombia; b Universidad Nacional de Colombia, Medellín, Colombia; c Universidad Pontificia Bolivariana (UPB), Medellín, Colombia; d Equal Opportunity Life Sciences (EQUOLS), Rockville, Maryland, USA

## Abstract

*Mycobacterium africanum* is a member of the *Mycobacterium tuberculosis* complex. Most commonly found in West African countries, it has scarcely been described in South America. Here, we report the first genome sequence of a Colombian *M. africanum* clinical isolate. It is composed of 4,493,502 bp, with 4,069 genes.

## GENOME ANNOUNCEMENT

*Mycobacterium africanum* is the causative pathogen of nearly half of the pulmonary tuberculosis cases in West Africa (1, 2). It has been characterized in 1968 in Senegal as an intermediate strain between *Mycobacterium tuberculosis* and *Mycobacterium bovis* (3). It consists of 2 phylogenetically distinct lineages within the *M. tuberculosis* complex, known as West African 1 and West African 2. The symptoms of infection caused by *M. africanum* are similar to those caused by its *M. tuberculosis* complex counterparts, and it is likely spread by aerosol transmission as well (4). Human tuberculosis caused by *M. africanum* has been reported in Africa, Australia, Europe, North America, and South America (3 – 6). However, no *M. africanum* isolate has been reported in Colombia to date.

Twenty-four-locus mycobacterial interspersed repetitive-unit–variable-number tandem-repeat (MIRU-VNTR) analysis was used to confirm that isolate UT307 belongs to the *M. africanum* lineage (7 – 9). An IS*6110*-based restriction fragment length polymorphism (RFLP) analysis revealed the presence of 4 copies of the insertion element. Spoligotyping showed the absence of spacers 7 to 9, 22 to 24, 35, 36, and 39.

Phenotypic susceptibility tests were performed using the Bactec MGIT 960 system. It was determined that UT307 is susceptible to streptomycin, isoniazid, rifampin, and ethambutol. Genomic DNA was obtained from isolate UT307 using the cetyltrimethylammonium bromide (CTAB) method (10). Whole-genome sequencing was performed at the BioFrontiers Institute Next-Generation Sequencing Facility (University of Colorado-Boulder), using an Illumina HiSeq 2000 platform with 100-bp reads and to about 228× coverage. *De novo* assembly was carried out using the SOAP*denovo*2 assembler (11), revealing a sequence length of 4,493,502 bp, with 65.1% G+C content. The *N*_50_ and mean contig lengths were 195,659 bp and 19,278 bp, respectively. The functional and structural annotations were completed with the Prokka software, which uses several sources of evidence, including the TIGRFAMs and Pfam protein family databases (12). The genome contains 4,018 coding sequences (CDSs) and 45 tRNAs. Large-sequence polymorphisms (LSPs) were evaluated, and we identified partial deletions of RD7, RD9, and RD10, and complete deletions of RD8 and RD702. Single-nucleotide polymorphisms (SNPs) Rv2427828GC and Rv378404GA (13) were identified, validating the assignment of isolate UT307 to the West African 2 lineage.

The whole-genome sequencing of UT307 confirms the circulation of *M. africanum* in Colombia. This first report will serve as a baseline further comparative genome analysis of *M. africanum* in Colombia and in the Americas.

### Nucleotide sequence accession numbers.

The genome sequence of the *M. africanum* isolate UT307 has been deposited at DDBJ/EMBL/GenBank under the accession no. CP014617. The version described in this paper is the first version, CP014617.1.
